# Supplementary figures and images for: Cluster analysis on high dimensional RNA-seq data with applications to cancer research - An evaluation study
Source: PLoS One. 2019 Dec 5;14(12):e0219102. doi: 10.1371/journal.pone.0219102 (PMC6894875; doi:10.1371/journal.pone.0219102)

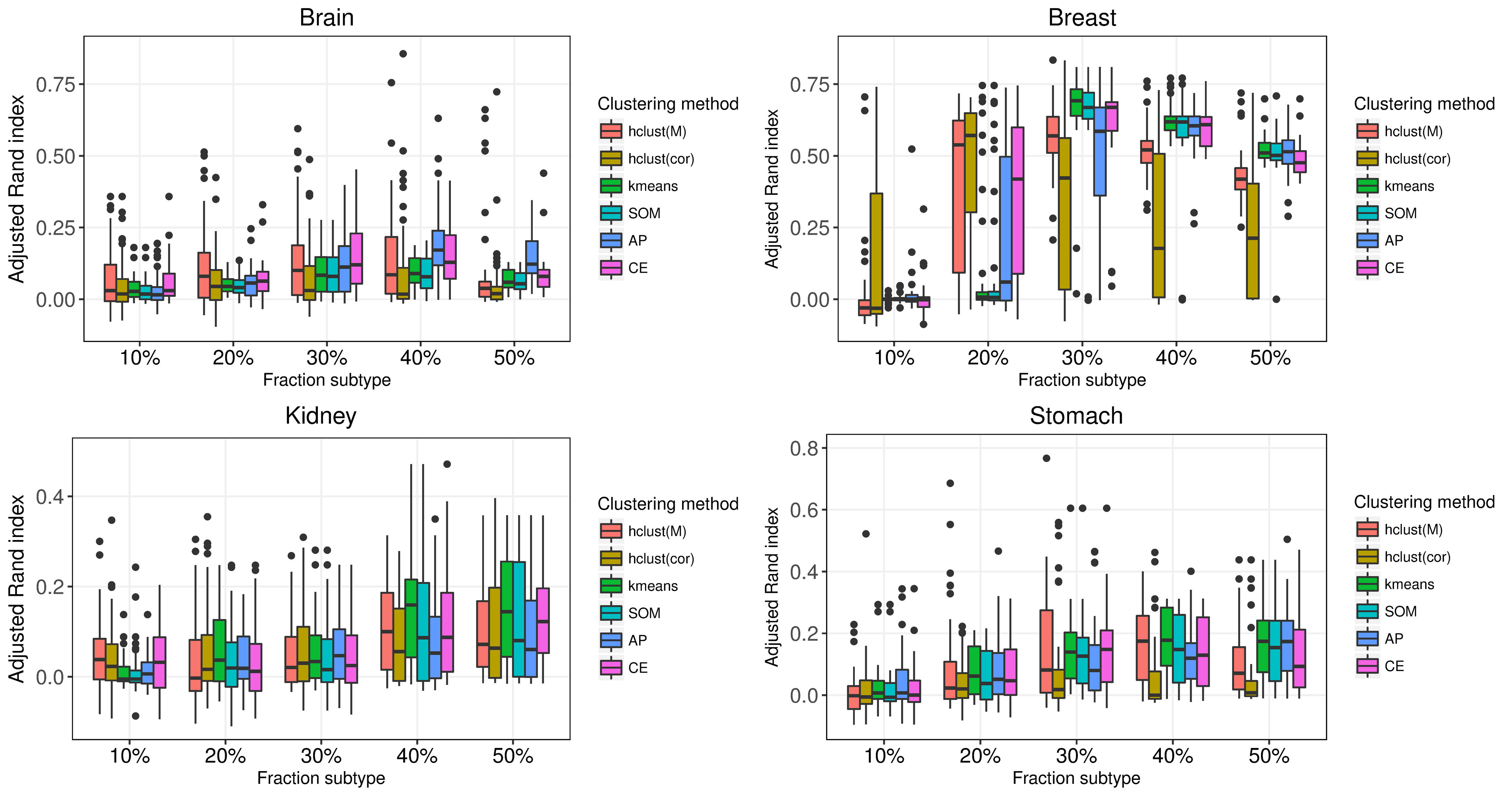

Supplement: S1 Fig — Adjusted Rand index for 10 random samplings and 5 gene selection methods. (TIF) [file pone.0219102.s015.tif]

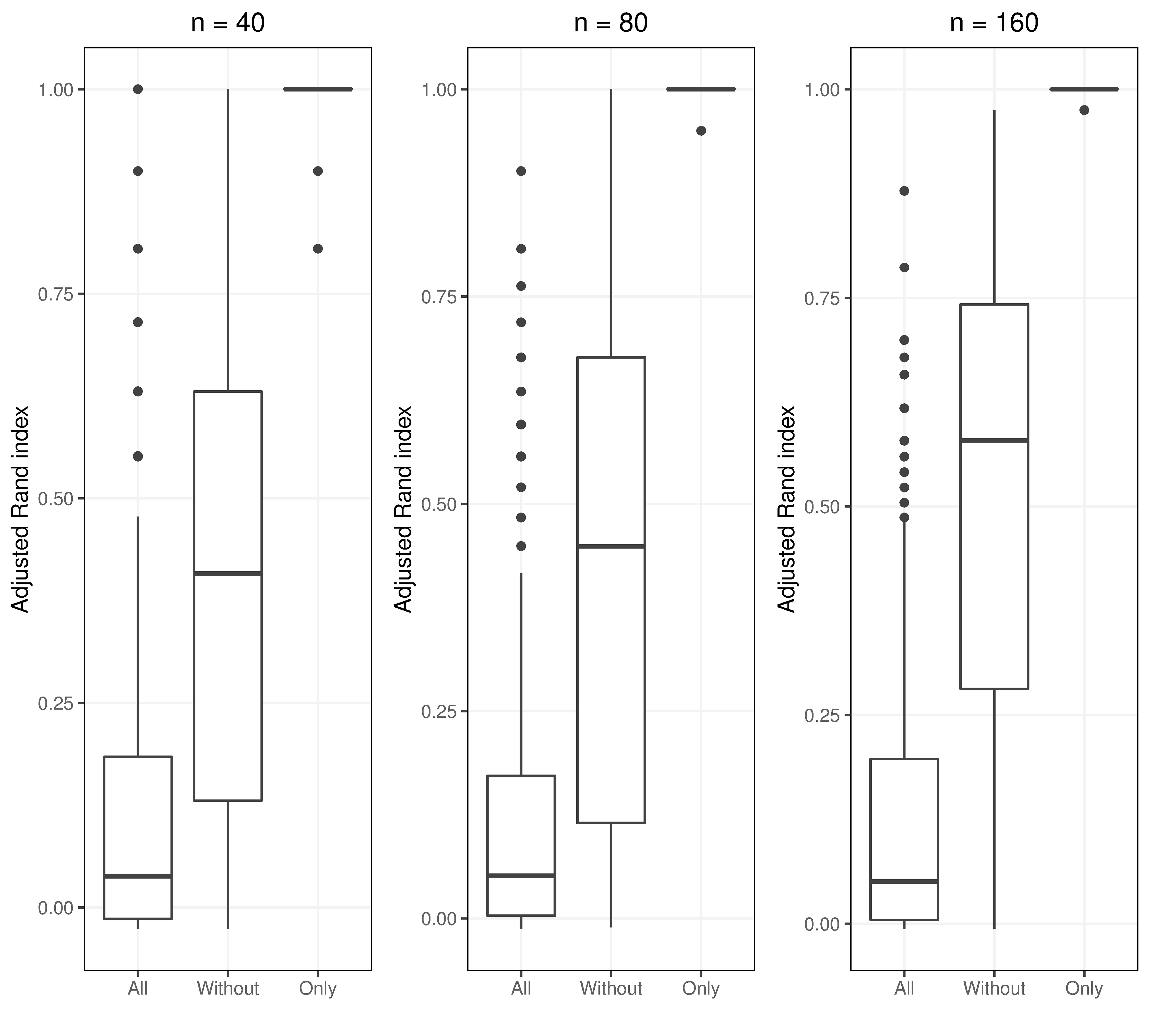

Supplement: S2 Fig — Thousand features (genes) were simulated for n patients (n = 40, 80, 160), where the patients were categorized with respect to gender, age (old or young) and cancer type (A or B). Fifty percent of the patients were male, 50% were young and 50% had type A such that the samples were divided in eight equally sized homogeneous groups. Twenty of the genes were affected by gender, 20 by age and 20 by cancer type and the remaining 940 genes were not affected by any factor. The expression values from the non-affected genes were simulated from a normal distribution with mean zero and standard deviation one (i.e. N(0,1)). The expression values of the affected genes were simulated from N(-1,1) if male/young/type A and N(1,1) if female/old/type B. Three hundred simulations were made for each considered sample size. Hierarchical clustering using the Manhattan distance and complete linkage was applied to each simulated sample. The resulting dendrogram was cut so that the samples were clustered into two groups and the results were compared to the partition defined by cancer type (i.e. the gold standard) using the adjusted Rand index. The clustering was made using all genes (All), excluding genes affected by either gender or age (Without) and including only the 20 genes affected by cancer type (Only). Here “All” relates to the problem when the data are affected by three factors, “Without” to the situation when the data are affected by a single factor, while “Only” relates to the ideal situation when only the cancer genes affect the clustering. Here the performance improvement when increasing the sample size was considerably lower for the complex problem (All) compared to the easier problem (Without). (TIF) [file pone.0219102.s016.tif]
